# Cold storage promotes germination and colonization of arbuscular mycorrhizal fungal hyphae as propagules

**DOI:** 10.3389/fpls.2024.1450829

**Published:** 2024-11-04

**Authors:** Xiaodi Liu, Guojian Ye, Zengwei Feng, Yang Zhou, Yongqiang Qin, Qing Yao, Honghui Zhu

**Affiliations:** ^1^ Key Laboratory of Agricultural Microbiomics and Precision Application (MARA), Guangdong Provincial Key Laboratory of Microbial Culture Collection and Application, Key Laboratory of Agricultural Microbiome (MARA), State Key Laboratory of Applied Microbiology Southern China, Institute of Microbiology, Guangdong Academy of Sciences, Guangzhou, China; ^2^ College of Horticulture, South China Agricultural University, Guangdong Province Key Laboratory of Microbial Signals and Disease Control, Guangzhou, China

**Keywords:** arbuscular mycorrhizal fungi, cold storage, propagules, hyphae, germination and colonization

## Abstract

The inoculants of arbuscular mycorrhizal fungi (AMF) propagated by the *in vitro* culture system is important in scientific research; however, the long-term storage reduces the spore germination rate. The propagules of AMF consist of three components, including spores, hyphae and colonized root fragments. It is well known that cold storage can improve the germination rate of AMF spores, with limited investigations on the germination of other propagules. In this study, AMF inoculants were stored at 25°C or at 4°C (cold storage) to investigate the effect of cold storage on the propagule viability of the AMF *Rhizophagus irregularis* DAOM197198. The germination rate of propagules (spores, hyphae, root fragments) and their colonization ability were determined at 3 and 6 months after storage. The results showed that the spore germination rate remained unchanged after storage for 0 and 1 month at 25°C, but decreased rapidly after storage for 3 months. Furthermore, we investigated the hyphal germination rate for the first time. The germination rates of spores, hyphae and root fragments were significantly higher under cold storage compared to those at 25°C. Additionally, we classified the germ tubes of hypha into two types: long-type (L-type) and short type (S-type). The germination rate and the proportion of L-type germ tubes of hyphae significantly increased with cold storage time, which was conducive to colonization. The results of mycorrhizal colonization confirmed that cold storage significantly increased the colonization of hypha compared with 25°C treatment. Cold storage may break the dormancy of AMF propagules and activate related enzymes to promote the germination and colonization of propagules, which needs further investigation.

## Introduction

Arbuscular mycorrhizal fungi (AMF) belong to Glomeromycotina, and can form mutually symbiotic relationships with the roots of more than 80% of terrestrial plant species (about 200,000 species) (Smith and Read, 2010; Spatafora et al., 2016; Jiang et al., 2021; di Fossalunga and Bonfante, 2023). Through the large hyphal networks of AMF, the host plant can obtain water and nutrients beyond the rhizosphere, especially phosphorus, which is difficult to move in the soil (Treseder, 2013; Yan et al., 2022). These symbiotic fungi also help host plants improve their ability to overcome stress, such as drought, salinity, heavy metals, low availability nutrients, extreme temperatures, acidic soils (low pH), aluminum toxicity, pollutants (arsenic and polycyclic aromatic hydrocarbons) and others (Cheng et al., 2021; Li et al., 2023; Razvi et al., 2023), which has great application potential in agricultural production (Berruti et al., 2016; di Fossalunga and Bonfante, 2023).

AMF are obligate biotrophs, and their spores reproduction rely on establishing symbiotic relationship with the host plants (Roth and Paszkowski, 2017; Feng et al., 2020). At present, there are two main ways to propagate spores: (1) Taking sorghum and/or clover (or other plant species) as host plants, propagate in mixture of soils, river sands, diatomaceous earth or other substrates (Fasusi et al., 2021; Corazon-Guivin et al., 2022); (2) Establishing an *in vitro* culture system with AMF and hairy roots (such as carrot and tomato), propagate under sterile conditions (Paré et al., 2022; Goh et al., 2022). Since the spores produced in the *in vitro* culture system are sterile, they are widely used in scientific research, production and other related fields (Diop, 2003; Calvet et al., 2013).

Spores, hyphae and colonized root fragments are all propagules of AMF that can be used as inoculants (Huey et al., 2020; Trejo-Aguilar and Banuelos, 2020; Vahter et al., 2023). The germination rate and the length of the germ tubes of propagules are important parameters to evaluate the quality of inoculants (Daniels et al., 1981; Fasusi et al., 2021), which determine whether the symbiotic relationship can be established, the level of symbiotic efficiency and the effect on plant growth (Barroso et al., 2022; Selvaraj and Thangavel, 2022). Therefore, improving the propagule activity of AMF plays a key role in effectively improving the quality of the inoculants. Spore germination may be affected by many factors, such as roots exudates, soil moisture, light exposure, temperature, pH, CO_2_, and flavonoids (Declerck and Angelo-Van Coppenolle, 2000; Bothe, 2012; Carvalho et al., 2015; Wang et al., 2015; Pei et al., 2020). Researches have confirmed that temperature can affect the germination rate of spores, with different effects across AMF species. The germination rate of *Glomus mosseae* spores increased after 5 weeks of culture at 6°C (Hepper and Smith, 1976). Juge et al. (2002) refrigerated the petri dishes with *Rhizophagus irregularis* DAOM197198 reproduction by *in vitro* culture system and found that storage at 4°C for two weeks or longer could promote the germination of spores from 50% to 90% and also increased the length of germ tubes. In contrast, the spore germination rates did not differ significantly between storage at 4°C and room temperature, and a lower temperature (-10°C for 4 weeks) was required to enhance spore germination of *G. mosseae* and *R. fasciculatus* (Safir et al., 1990). However, long-term frost (-20°C) storage reduced the colonization capacity of AMF (Püschel et al., 2019), even for native fungi derived from arctic frozen soils.

Previous works on propagule germination mainly focused on spores, while hyphae and colonized root fragments, which are also important propagules of AMF, have received little attention. Therefore, in this study we used *R. irregularis* DAOM197198 as the test AMF, which is the most commonly used isolate for *in vitro* culture system in scientific studies. The hairy roots of tomato (*Solanum lycopersicum* cv. Xinjinfeng) were used as the host plant. Monoxenic cultures of AMF spores and hairy roots at 25°C for 3 months were used as the initial material (0 month), and two treatments with contrasting storage temperatures were set up, namely, 4°C refrigeration and 25°C incubation. The germination, colonization and sporulation ability of different propagules (spores, hyphae and root fragments) were measured after 0, 1, 3 and 6 months of treatment to explore whether cold storage can improve the germination rate of propagules, and then improve the colonization and sporulation ability of propagules.

## Materials and methods

### Biological material

Tomato (*Solanum lycopersicum* cv. Xinjinfeng) hairy roots transformed with *Agrobacterium rhizogenes* ACCC 10060 (GDMCC 1.753) according to the method by Bécard and Fortin (1988) was used as host plant. *R. irregularis* DAOM 197198 (GDMCC 3.720) was used as the AMF material. Propagules of the AMF were obtained from *in vitro* monoxenic cultures with tomato roots maintained on modified Strullu-Romand (MSR) medium (Bécard and Fortin, 1988) in Petri dishes [0.25% (w/v) Phytagel from sigma] at 25°C in an incubator.

### Experimental setup and sampling

Petri dishes containing spores, roots, and hyphae after three months of *in vitro* culture of tomato roots and AMF were used in this experiment. To measure the influence of different culture conditions on propagule germination, 60 petri dishes were prepared. Randomly selected 30 petri dishes continued to cultivate in the incubator at 25°C while the left 30 petri dishes were refrigerated at 4°C. After incubation at 25°C or refrigeration at 4°C for 0, 1, 2, 3 and 6 months respectively, samples were taken and the propagules were collected for further test.

To collect the propagules in each petri dish, the medium was dissolved with sodium citrate buffer of pH 6.0, and the spores and hyphae entangled on hairy roots were picked out with two tweezers under the microscope in a benchtop. Subsequently, the tangled spores and hyphae were also separated under the microscope. Then the hyphae were cut into a length greater than 2.5mm under the microscope, which could be used as the hyphae inoculation material. In addition, the spores attached to the hyphae were picked off one by one to serve as the inoculation material for the spores. After removing all the hyphae and spores from the plate, the roots were dissected into 1cm fragments using sterilized scissors, which served as inoculation material for the root segments.

The three types of propagules were inoculated on the MSR medium in a uniformly dispersed manner. Each petri dish was inoculated with 100 spores, 50 hyphae or 100 root fragments, respectively. The petri dishes were sealed with sealing film and placed in an incubator for dark culture at 25°C.

In order to investigate the viability of different propagules, the ability of different propagules to colonize roots and reproduce spores was assessed. Petri dishes stored at 25°C (T1) and 4°C (T2) for up to 6 months were selected as the treatment group, with the initial petri dishes as the control (CK). Each treatment had 5 replicates. The sampling method of spores and hyphae was the same as above. On MSR plates, 50 spores or 50 hyphae were inoculated, and 5 hairy roots of tomato 3-4 cm were placed on each plate, sealed with sealing film and cultured in an incubator at 25°C for 3 months, and the mycorrhizal colonization of spores and hyphae in different treatments was determined.

### Determination of propagule germination rate

After incubation for 3 weeks, the germination of spores, hyphae and root fragments were observed under the microscope, and the germination rates of different propagules was counted.

### Measurement of the germ tube length of spores and hyphae

Twenty-five germinated spores and twenty-five germinated hyphae were randomly selected, photographed under a stereomicroscope. Then the length of each spore and hyphal germ tubes was measured by using ImageJ software (https://imagej.nih.gov/ij/). The proportion of germ tube longer than 1 mm in germinated hyphae was calculated as: 100% × the number of hyphal fragments with germ tube longer than 1 mm/the total number of germinated hyphal fragments.

### Determination of mycorrhizal colonization and the number of spores

Mycorrhizal colonization was determined according to the method of Phillips and Hayman (1970). Briefly, about one hundred fine root fragments were added to a 2 mL centrifuge tube containing 1.5 mL of 5% KOH solution, and incubated in a water bath at 90°C for 30 min, then bleached with 10% alkaline hydrogen peroxide solution for 15 min, acidified in 2% HCl solution for 10 min at room temperature, and then stained with 0.05% trypan blue at 90°C for 30 min. Mycorrhizal colonization was quantified according to the method of Trouvelot et al. (1986) with the software MYCOCALC (https://www2.dijon.inrae.fr/mychintec/Mycocalc-prg/download.html). When spores were used as inoculants, 30 randomly selected root segments were chosen for each sample, while when hyphae were used as inoculants, 30 randomly selected root segments were chosen for each sample, and placed them on a microscope slide (Olympus BX53) for observation. F% (the colonization frequency), M% (the colonization intensity), m% (the relative colonization intensity), A% (the arbuscular abundance) and a% (the relative arbuscule abundance) were calculated with MYCOCALC. Number of spores was observed and recorded under microscopy (Olympus BX53).

### Statistical analysis

All data are presented as the mean ± standard error of more than 5 replicates. All treatments and parameters were tested for normal distribution before using other statistical methods. Analysis of variance (ANOVA), Two-way ANOVA, Tukey’s honestly significant difference (Tukey’s HSD) test, Independent sample t test were performed with SPSS statistical software (v21.0, SPSS Inc., Chicago, IL, USA). Significance was assessed at *P* < 0.05.

## Result

### Cold-storage promoted the germination of propagules

**Figure 1:**
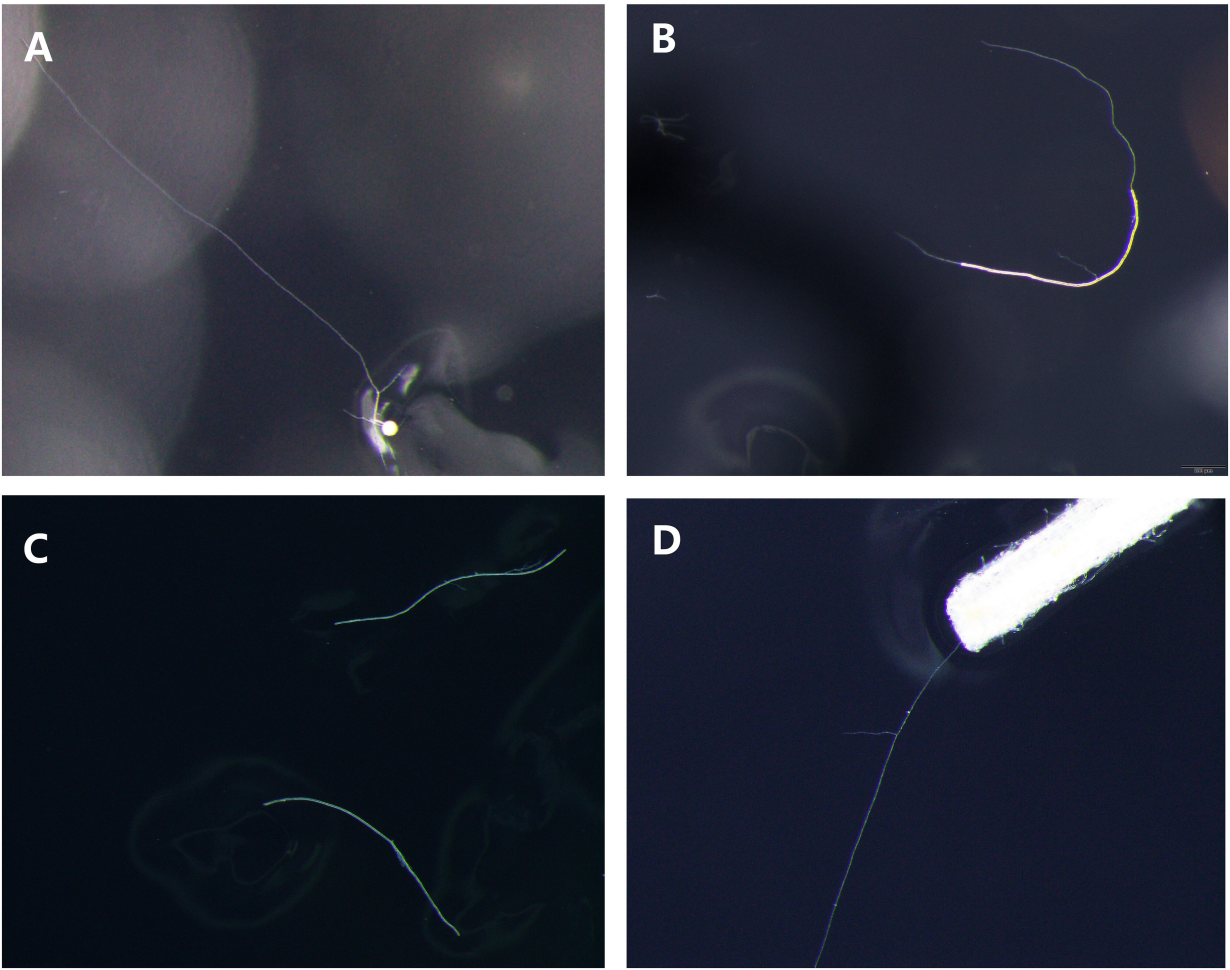
Germination morphology of different propagules. **(A)** germinated spore; **(B)** germinated hypha; **(C)** non-germinated hyphae; **(D)** germinated roots.

The germination state of different propagules of *R. irregularis* DAOM 197198 from *in vitro* monoxenic cultures was observed, and the pictures of germinated spore (**Figure 1A**), germinated hypha (**Figure 1B**), non-germinated hyphae (**Figure 1C**) and germinated roots (**Figure 1D**) were shown in **Figure 1**. For the first time, the germination morphology of hyphae was observed under a stereomicroscope (**Figure 1B**), although previous reports have speculated that hyphae could be used as inoculants to colonize roots. Simultaneously, the germination morphology of colonized root fragments was also observed, and the germ tubes extended from the broken root segment (**Figure 1D**).

**Figure 2:**
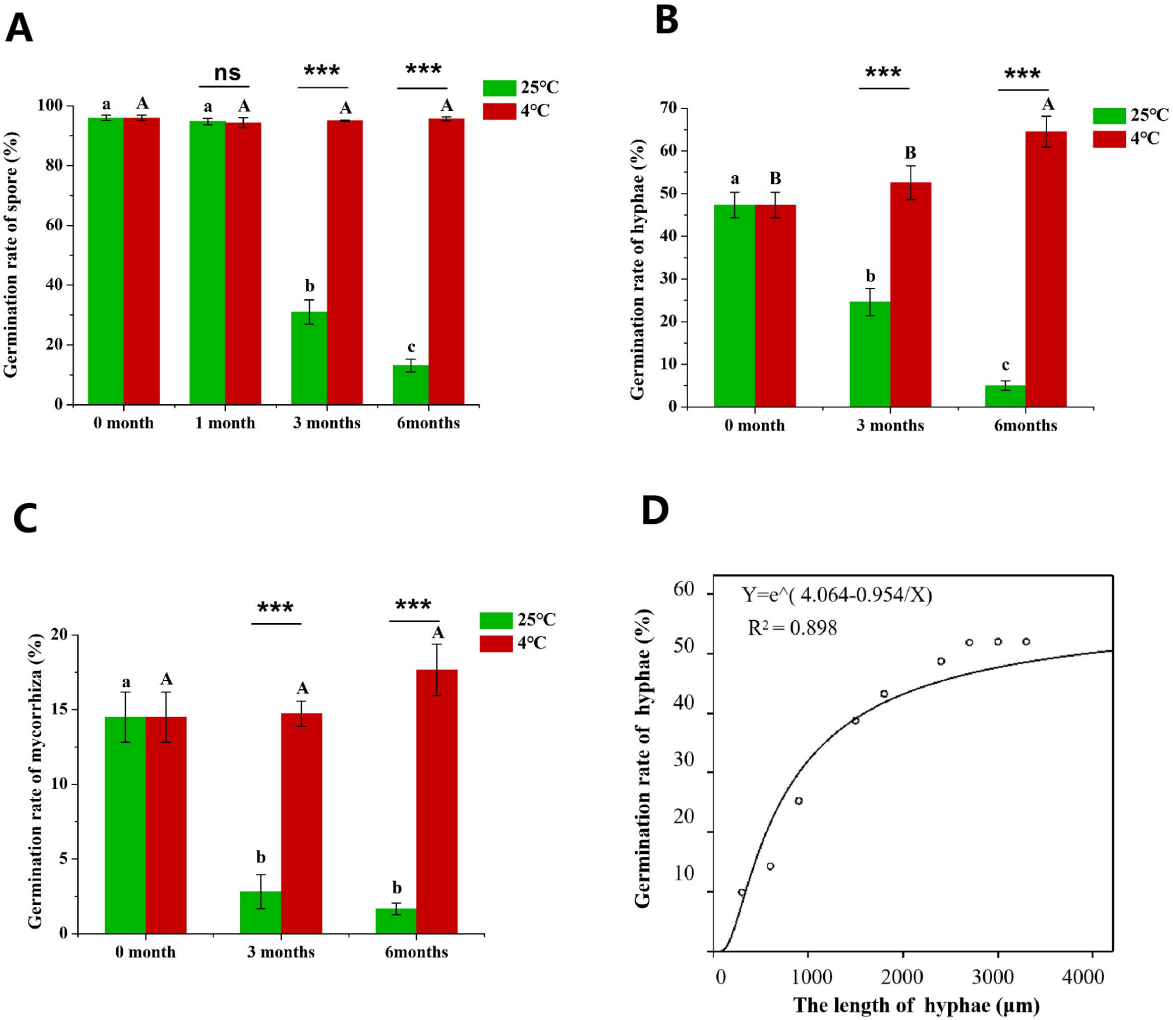
The effect of long-term culture of AMF in *in vitro* culture system on the germination of propagules. **(A)** Effects of different refrigeration time on spore germination rate (Sgr), **(B)** effects of different refrigeration time on hyphae germination rate (Hgr), **(C)** effects of different refrigeration time on roots germination rate (Rgr). **(D)** the fitting curve between Hgr and hyphae length. Different lowercase letters indicate significant difference between different storage time at 25°C, and different uppercase letters indicate significant difference between different refrigeration time at 4°C. “***” indicate that the germination rate between different storage temperatures is significant difference (***P < 0.001), and “ns” indicates that there is no significant difference between different storage temperature treatments.

The germination rate of *R. irregularis* propagules including spores, hyphae and root fragments were determined. We found that the germination rate of hyphae (Hgr) was correlated with the length of hyphae fragment, so we put forward a fitting curve to illustrate the function relationship between Hgr and hyphal length (**Figure 2D**). The results showed that the Hgr rose with the increase of hyphae length. The Hgr remained at about 50% when the hyphae length was more than 2.5 mm (**Figure 2D**). Therefore, the hyphae were divided into fragments of more than 2.5 mm for inoculation, when the germination rate were measured.

Sterilized propagules were collected from *in vitro* monoxenic cultures of *R. irregularis* in association with tomato hairy roots grown on MSR medium at 25°C in the dark for 3 months. At this time, the germination rate of propagules was taken as the initial value (0 month). The initial germination rate of spores (Sgr) of *R. irregularis* was as high as 96.0% (**Figure 2A**). The Hgr was 47%, which was about half of the spores (**Figure 2B**). The germination rate of root fragments (Rgr) was 14.5%, which was the lowest among the three types of AMF propagules (**Figure 2C**).

When stored at 25°C, there was no significant difference in spore germination rate between 1 month and 0 month (**Figure 2A**), so the Hgr and Rgr were not detected at 1 month. With the increase of cultivation time, the Sgr was significantly inhibited (**Figure 2A**), and the Hgr and Rgr were also significantly inhibited (**Figures 2B, C**). The germination rate of spores, hyphae and root fragments at 3 months of culture were significantly lower than the initial values (**Figures 2A–C**). The Sgr and Hgr at 6 months were significantly lower than 3 months (**Figures 2A, B**). At 3 months, the germination rates of spores, hyphae and root fragments were 31.0%, 24.6% and 2.8%, which were 67.7%, 48.1% and 80.7% lower than the initial values (**Figures 2A–C**). At 6 months, the germination rates of spores, hyphae and root fragments were 86.4%, 89.5% and 88.3% lower than 3 months (**Figures 2A–C**). The longer the storage time, the lower the germination rate of different propagules. In conclusion, long-term *in vitro* monoxenic culture at 25°C in incubator inhibited the germination of AMF propagules.

Considering that cold-storage has been usually used to preserve AMF inoculants, in this study, we investigated the effect of cold-storage on the germination rate of propagules. Six months after cold-storage (MAC), the Sgr was remained at 95%, which was close to the initial Sgr (**Figure 2A**). The Hgr rose with the increase of cold-storage time (**Figure 2B**). The Hgr at 6 MAC was significantly higher than the initial value (**Figure 2B**). Similarly, the Rgr also rose with the increase of cold-storage time (**Figure 2C**). In the cold-storage treatment, the germination rates of spores, hyphae and root fragments were significantly increased at 3MAC and 6 MAC compared with culture at 25°C (**Figures 2A–C**). The germination rates of hyphae and roots were lower than that of spores, and the germination rate of different propagules showed the following rule: spores > hyphae > roots (**Figures 2A–C**). Finally, we are concerned that cold storage could maintain the germination rate of spores and root fragments in AMF propagules, and significantly promoted the germination of hyphae compared to storage at 25°C.

### Cold storage promoted the germination and elongation of hyphae

**Figure 3:**
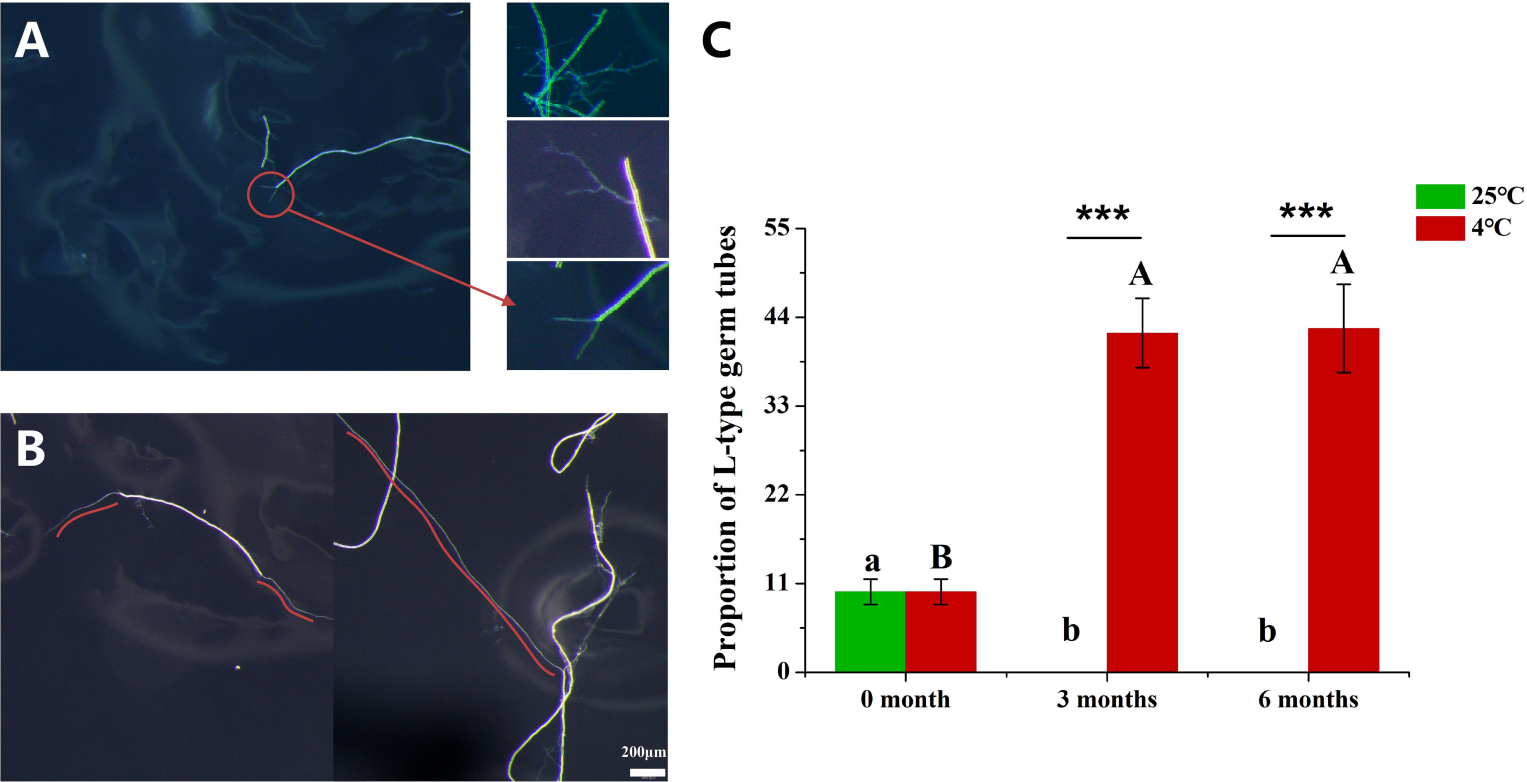
The proportion of hyphae with L-type germ tubes to total hyphae. **(A)** The germ tube is short and thin, and the length is generally less than 0.5mm. **(B)** The germ tube is long and thick, the length is greater than 0.5mm, or even greater than 1cm. **(C)** The proportion of hyphae with L-type germ tubes to total hyphae. The red curve shows the growth trajectory of the germ tubes. “***” above graphs indicate that the length of L-type germ tube significant differences with different storage temperatures using Independent samples t-test (***P < 0.001). Distinct uppercase and lowercase letters indicate a statistically significant difference at 4 °C and 25 °C.

We observed the germination of hyphae, and found that there were two distinct types of germ tubes mainly according to their lengths. We defined these two types of germ tubes as S-type (short-type) (**Figure 3A**) and L-type (long-type) (**Figure 3B**). In S-type germ tubes, it was observed that one hypha produced one, two, multiple or highly branched clusters of germ tubes, all of which had a common feature, that is, the longest germ tubes were about 0.1-0.2 mm (**Figure 3A**). The L-type germ tubes were characterized by a clearly distinguishable main hypha, and all the germ tubes were longer than 0.5 mm and even up to 20 mm length (**Figure 3B**). At 0 month, L-type germination accounted for about 10% of the total hyphae inoculated in the plates, while the proportion of L-type germination was 42% which was significantly higher than the initial value at 3 MAC and 6 MAC (**Figure 3C**). In addition, we observed that a small amount of hyphae could produce two or three long germ tubes above 0.5 mm length at 6 MAC (**Supplementary Figure S1**). By contrast, no L-type germination was observed 3 and 6 months after 25°C culture. The above results indicate that cold storage promoted the growth of germ tubes of the hyphae. In addition, the previous results in this study showed that cold-storage promoted the germination of hyphae (**Figure 2B**). In conclusion, cold storage not only increased the germination of hyphae, but also enhanced the growth of hyphae.

### The germ tubes of hyphae are shorter than those of spores

**Figure 4:**
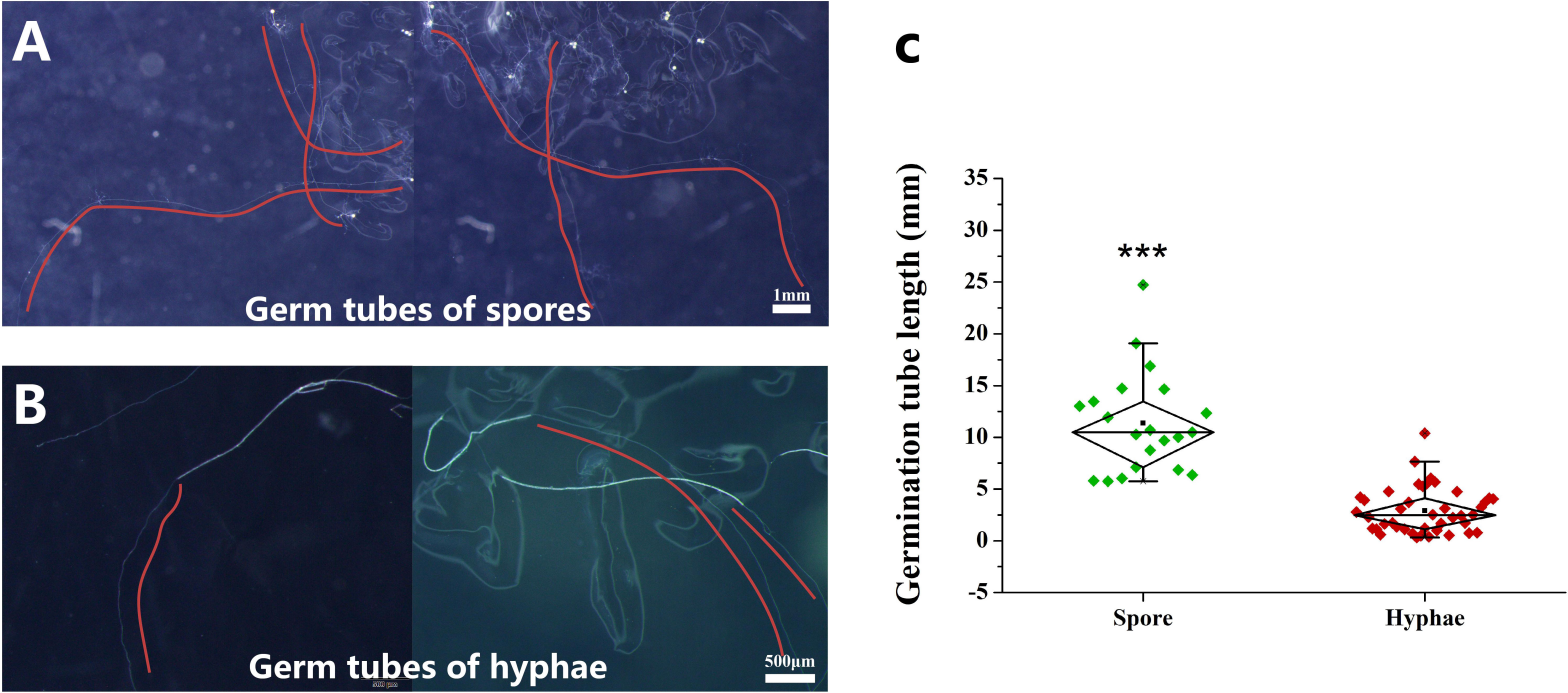
Germ tube length of spores and hyphae after 6 months of cold storage at 4°C. **(A)** The germ tubes of spores, **(B)** the germ tubes of hyphae, **(C)** the difference in the length of germ tubes between spores and hyphae. The red curve shows the growth trajectory of the germ tubes. “***” indicates that there is a significant difference in the length of the germinating tubes between the spores and the hyphae using Independent samples t-test (***P < 0.001).

During the experiment, we observed that the germ tubes of hyphae were much shorter than those of spores, although cold storage significantly promoted the growth of the germ tubes of hyphae (**Figures 4A, B**). We analyzed the difference of germ tube length between spore and hyphae at 6 MAC. The results showed that the longest germ tubes of hyphae was about 1 cm, and the longest germ tubes of spores could reach 2.5 cm (**Figure 4C**). T-test analysis showed that the length of germ tubes of spores was significantly longer than that of hyphae (**Figure 4C**). According to the above results, we speculate that the colonization ability of hyphae on host roots was lower than that of spores.

### Cold-storage promoted the mycorrhizal colonization of AMF propagules

We found that some hyphae were attached to the roots and was difficult to distinguish, though we have tried our best to pick out the hyphae on the roots, when the Rgr was measured. The roots used to determine Rgr were divided into two groups: germinated roots and non-germinated roots, then we measured the mycorrhizal colonization of two groups of roots.

As shown in **Table 1**, the mycorrhizal colonization of germinated roots was 64.3%, while no colonization structure was observed in 35.7% of germinated roots (**Table 1**). Since some hyphae may be wrapped around some roots, the colonization and sporulation capacity of the roots could not be accurately counted. Therefore, only the colonization ability of spores and hyphae was counted, which was sufficient to reflect the quality of inoculants.

**Table 1:** Mycorrhizal colonization of germinated and non-germinated roots.

	Mycorrhizal colonization	Root with endospore/Mycorrhizal
GR	64.3%	33.3%
NGR	14.3%	28.6%

GR, germinated roots; NGR, non-germinated roots. Dissolve the inoculants cultured in the *in vitro* culture system for 3 months with sodium citrate buffer (pH 6.0). The roots were cut into 1cm fragments, and the hyphae and spores wound around the roots were picked out under a stereomicroscope. Then 15 MSR plates were prepared, and 100 root fragments were placed on each plate. The plates were sealed with parafilm and placed in a dark incubator at 25°C. 50 germinated and non-germinated root fragments were collected and stained with trypan blue to measure the mycorrhizal colonization.

**Figure 5:**
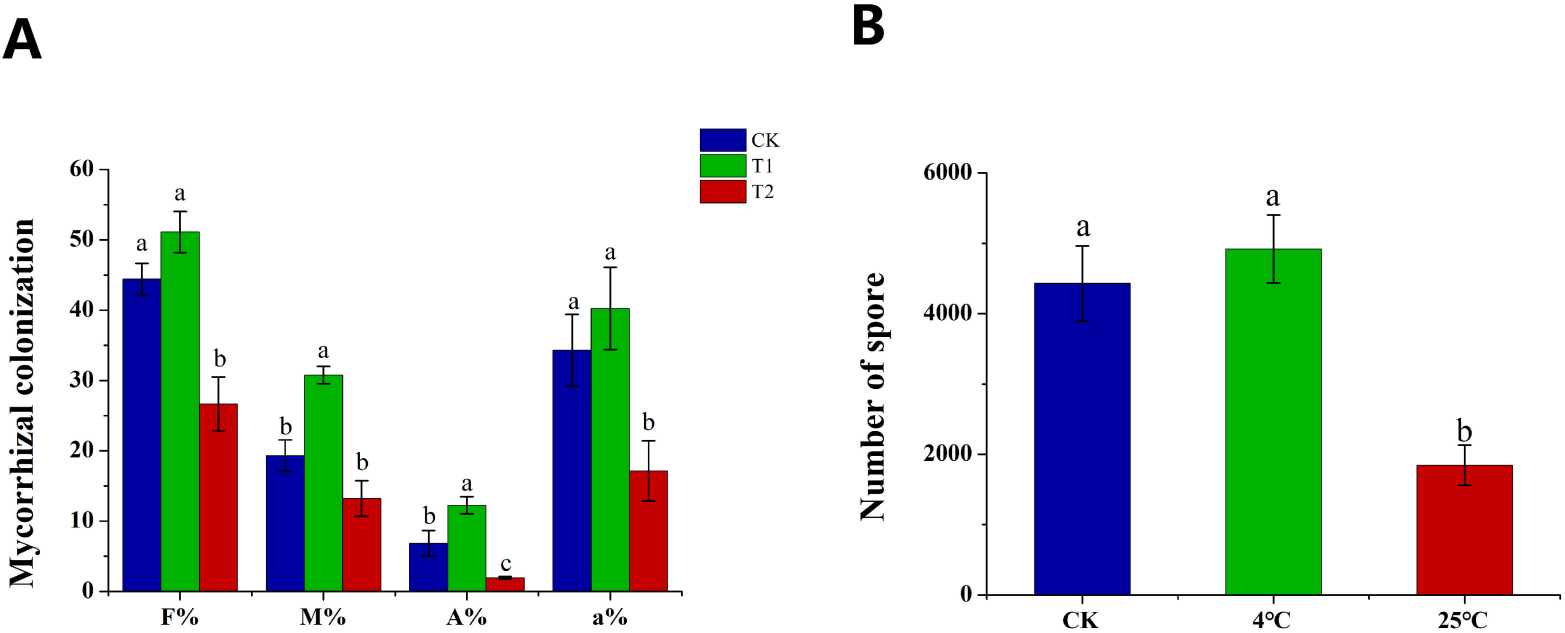
The effect of spores refrigeration for 6 months on mycorrhizal colonization and sporulation. **(A)** Effect of spores as inoculants on mycorrhizal colonization, **(B)** Effect of spores as inoculants on sporulation. CK: spores under initial conditions, T1: spores storage at 25°C for 6 months as inoculants, T2: spores refrigeration at 4°C for 6 months as inoculants. F%: colonization frequency, M%: colonization intensity, A%: arbuscular abundance, a%: relative arbuscular abundance. Different lowercase letters indicate significant differences between treatments (*P* < 0.05).

Hyphae and spores in the plates of *in vitro* cultured AMF preserved at 25°C (T1) and 4°C (T2) for 6 months were used as inoculants to establish symbiotic relationship with tomato hairy roots. The plates of 0 month were used as the control (CK). With spores as inoculants, we evaluated the mycorrhizal colonization based on four parameters, e.g., colonization frequency (F%), colonization intensity (M%), arbuscular abundance (A%) and (the relative arbuscular abundance) a%. The F%, A% and a% of T1 were significantly lower than those of the control, by contrast, there was no significant difference in F% and a% of T2 compared with the control (**Figure 5A**). To our excitement, the M% and A% of T2 increased significantly compared with the control (**Figure 5A**). In addition, the four mycorrhizal colonization parameters of T2 were all significantly higher than those of T2. Similar to the results of mycorrhizal colonization, the sporulation of T1 was significantly lower than that of the control and T2, while there was no significant difference between T2 and the control (**Figure 5B**). The results indicated that long-term culture at 25°C significantly inhibited the colonization and sporulation ability of spores, which could be maintain and even promote by long-term cold storage.

**Figure 6:**
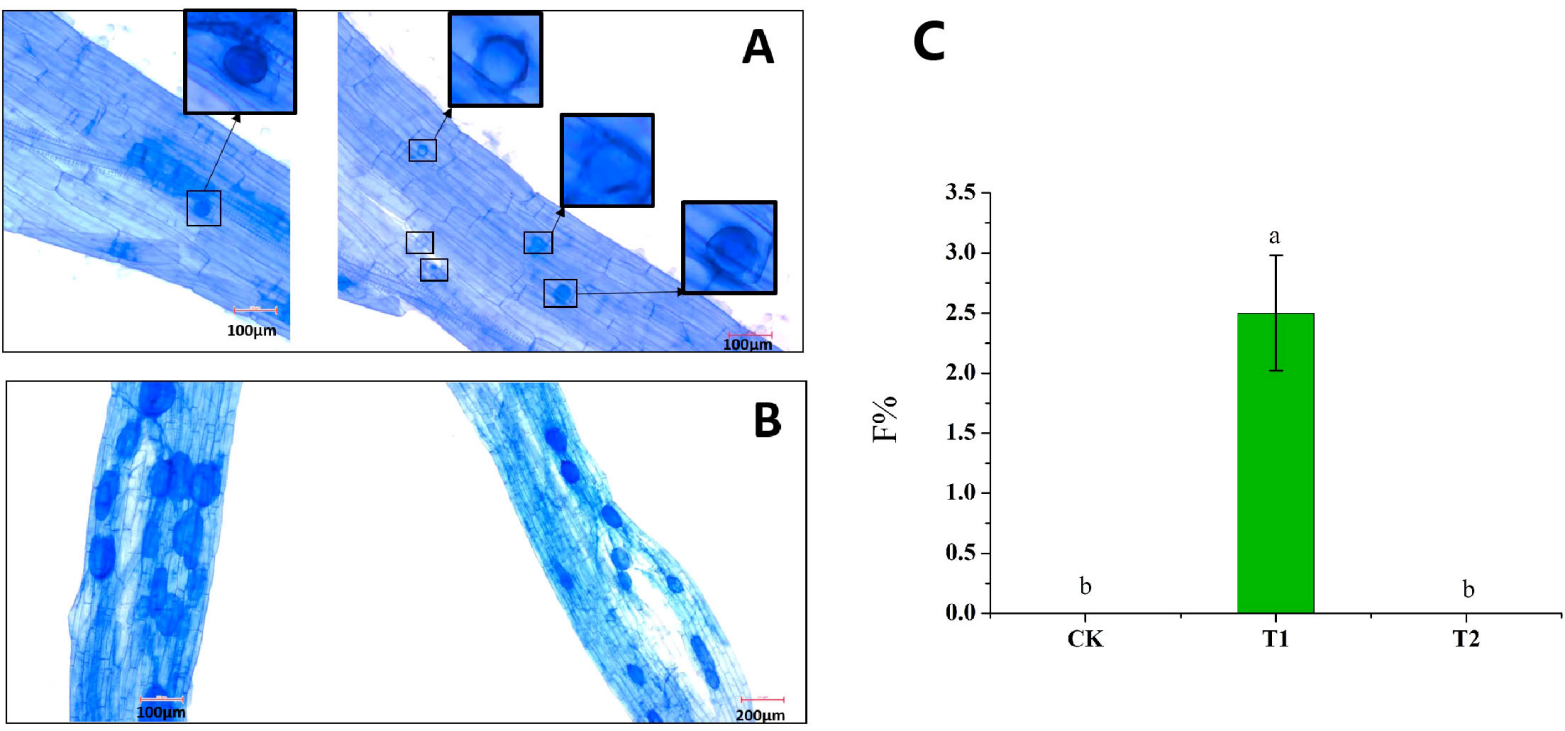
Effect of hyphae refrigeration for 6 months on mycorrhizal colonization. **(A)** Mycorrhizal colonization pictures with hyphae as inoculants after 6 months of refrigeration. The black boxes represent the endospores, and the red boxes were the larger images of the black boxes. **(B)** Mycorrhizal colonization pictures with spores as inoculants after 6 months of refrigeration. **(C)** The colonization frequency of hyphae. CK: the hyphae of the initial condition T1: the hyphae stored at 25°C for 6 months were used as the inoculants, T2: the hyphae stored at 4°C for 6 months were used as the inoculants. Different lowercase letters indicate significant differences between treatments (*P* < 0.05).

With hyphae as inoculants, we observed that hyphae were able to infect roots for the first time; however, arbuscule was not observed. Therefore, only colonization frequency (F%) was assessed. The F% of T2 was significantly higher than that in CK and T1, indicating that cold storage significantly promoted the colonization of hyphae to roots (**Figure 6C**). No colonization was observed in CK and T1 treatments, and only a small amount of colonization was observed in T2 (**Figure 6C**). Intraradical spores could also be observed in the root fragments in T2 (**Figure 6A**), while extraradical spore formation was not observed. Compared with spores as inoculants, mycorrhizal colonization of hyphae as inoculants was lower (**Figures 5A**, **6C**), which was consistent with our previous speculation. When hyphae are used as inoculants, the intraradical spores were fewer and smaller, and the staining color of Trypan blue was lighter than that of spores as inoculants (**Figures 6A, B**).

## Discussion

There are two common propagation methods of AMF. One is the potted propagation method, with which AMF propagules are inoculated into the potted substrate grown with host plants (Lalaymia et al., 2013; Liu et al., 2020). The other is the dual-culture propagation method, with which AMF spores are inoculated to Ti-plasmid transformed hairy roots in *in vitro* culture system to obtain aseptic inoculants (Bécard and Fortin, 1988; Lalaymia et al., 2013, 2014; Liu et al., 2023).

In this study, long-term room temperature culture significantly decreased the germination rate of AMF spores, while cold storage could maintain a higher level of spores germination rate, and significantly increased the germination rate of spores compared with room temperature culture. Compared with 0 month, cold storage did not significantly increase the germination rate of spores, probably because the germination rate of spores in our experiment was as high as 96% at 0 month, which was similar to the highest germination rate of spores in Juge et al. (2002).

There are many studies on the preservation of AMF inoculants for potted culture and propagation. The spore germination rate of AMF and other filamentous fungi were reduced when the inoculants were stored at room temperature for long term. Wagner et al. (2001) investigated the effect of storage conditions on *Glomus claroideum*, and the inoculants were store at 4°C and 24°C for 272 weeks. The number of spores and propagule activity were assessed by sucrose gradient centrifugation and most probable number methods. After 272 weeks of storge, the number of propagules stored at 24°C was 10/g, and the number of propagules refrigerated at 4°C was 300/g, which was 30 times that of stored at 24°C. The number of extracted spores from propagule was 50 for cultures stored at 4°C but less than 5 spores/g at 24°C. The results showed that the spore viability decreased exponentially with storage duration, and refrigeration at 4°C significantly promoted the number and viability of spores. Püschel et al. (2019) measured the infectivity of AMF inoculants by mixing the intraradical fungus inoculants with peat soil and storing at -20, 5 and 20°C, and found that with the increase of culture time, the infectivity at 20°C decreased sharply, and the infectivity of AMF inoculants stored at 5°C remained unchanged or even increased during the experiment. In addition, the number of propagules, the number of spores and the colonization ability of AMF of different species were different. AMF plates cultured for 2-3 months were refrigerated for 0, 3, 7, 14, 90, or 120 days at 4°C. Cold storage significantly increased the germination rate of spores, reduced the spore mortality and significantly changed the hyphae growth pattern (Juge et al., 2002). Previous studies supported our results on the difference in AMF spore viability under different preservation conditions.

In addition, we investigated the effect of different treatments of spores on mycorrhizal colonization, and found that cold storage significantly increased the mycorrhizal colonization of spores. Compared with the incubation at 25°C for 6 months, cold storage significantly promoted the mycorrhizal colonization capacity of spores. In addition, the M% and A% of the cold storage treatment were significantly higher than the initial value (0 month). In general, long-term preservation can reduce the germination rate and colonization ability of spores, and cold storage can promote the germination and colonization of spores. The germination ability of spores determines the mycorrhizal colonization ability of spores.

The decrease of spore germination rate after long-term cultivation at room temperature may be attributed to the DNA damage and a reduction in enzyme activity within the spores. Bainard et al. (2010) investigated the effects of different preservation methods on the quality and yield of DNA extraction from Bromus inermis and Daucus carota colonized by the AM fungus *Glomus intraradices*. Compared with cryopreservation, the concentration of DNA in mycorrhiza was significantly reduced (*Bromus inermis*) or undetectable (Carrot) after 15 weeks of incubation at 37°C. Moreover, we observed the growth state of symbionts in *in vitro* culture system (**Supplementary Figures S2, S3**). Compared to storage at 25°C for 6 months, storing the spores and hyphae at 4°C for the same duration resulted in enhanced germination rates and longer germ tube lengths (**Supplementary Figure S2**). Conversely, after being stored at 25°C for 6 months, both spores and hyphae exhibited lower germination rates, shorter germ tubes, and minimal or no germination occurred (**Supplementary Figure S2**). The length of the germ tube directly influences the mycorrhizal colonization ability of propagules. Cold storage could maintain or promote the length of propagule’s germ tube, thereby further facilitating mycorrhizal colonization. The spores remained milky white at 4°C for 6 months, which was similar to that of 0 month. There was no significant difference between the growth state of roots storage at 4°C for 6 months and the initial state (**Supplementary Figure S3**). However, the spores cultured at 25°C for 6 months turned yellow, and the roots were obviously senescent compared to those at 0 month (**Supplementary Figure S3**). Cold storage may induce a reduction in metabolism to sustain the heightened activity of AMF propagules, or it may break the dormancy of spores in AMF and subsequently promote spore germination and root colonization.

Numerous reports have proposed that the spores, hyphae, and colonized root fragments can be used as propagules of AMF. The majority of studies investigating propagule viability have primarily focused on spores, examining factors such as temperature, pH, nitrate levels, and heavy metal concentrations in relation to spore germination rates and colonization on roots. Hyphae are an important propagule of AMF, but has been little research on their germination ability. In our study, we observed hyphae germination for the first time, and we also observed the germination of root fragments, which is consistent with what was observed by Diop (2003). The germ tubes of hyphae were formed from the break of hyphae, and each hypha can form one, two or multiple germ tubes.

When we calculated the germination rate of root segments, 35.7% of the germinated roots showed no colonization structure. It suggested that the germ tubes of root segments were not produced from the infecting structures within the root entirely, but partly from the hyphae surrounding the roots. In roots with spores or vesicles, the intraradical hyphae or arbuscules must be observed. In theory, spores could germinate, but it was unclear whether vesicles could germinate. Intraradical spores or vesicles were observed in 33.3% of colonizad roots, and only hyphae or arbuscules could be observed in 66.7% of colonizad roots (**Table 1**). The result implied that both hyphae and arbuscules had the ability to germinate. The mycorrhizal colonization of non-germinated roots was 14.3% (**Table 1**), indicating that although these roots were colonized, they had no ability to germinate. In addition, more direct evidence for the germination of the colonized structures in the roots are needed.

Cold storage not only significantly increased the germination rate of hyphae, but also significantly promoted the proportion of L-type germ tubes, that is, promoted the length of hyphal germ tubes. Cold storage can promote the germination of spores (Safir et al., 1990; Juge et al., 2002; Püschel et al., 2019), but there is no research on the effect of cold storage on hyphae germination. The work conducted by Juge et al. (2002) demonstrated that the activation of spore germination could be achieved when the *in vitro* cultured *Glomus intraradices* spores were stored at 4°C for at least 2 weeks. The dormancy of spores, even in controlled laboratory conditions, is likely regulated by an intrinsic biological clock. Cold stratification may not be essential for breaking dormancy; however, it has the potential to trigger or expedite associated physiological modifications (Juge et al., 2002). The utilization of cold storage can facilitate the disruption of spore dormancy, enhance spore germination, and augment colonization capability. Nevertheless, Cold storage induced hyphae germination mechanism remains unclear, potentially involving the same principles as spore dormancy breaking or other factors.

For the first time, we observed the colonization of roots by hyphae as propagules The initial hyphae (0 month) and the hyphae stored at 25°C for 6 months were co-cultured with tomato hairy roots as propagules, and no colonization was observed in the roots. Mycorrhizal colonization was observed when the hyphae stored at 4°C for 6 months were used as propagule. Cold storage significantly enhanced the colonization of hyphae on roots, because it promoted both the germination rate and growth of hyphal germinating tubes, thereby facilitating the colonization of hyphae on roots. Although cold storage promoted mycorrhizal colonization of hyphae, the ability of mycorrhizal colonization was significantly lower than that of spores. The colonization rate of hyphae as propagules was approximately 2.5%, which was much lower than that of spores as propagules (53%). In addition, we observed that the hyphae as propagules formed intraradical spores, which were smaller than that of intraradical spores formed by spores as propagules. However, neither arbuscular nor extraradical spores were observed. It is possible that increasing the amount of inoculated hyphae may lead to the observation of more intraradical or extraradical structures, or it could be that hyphae as inoculants do not form arbuscular and extraradical spores, which requires further study. The inoculated propagule in the 25°C treatment and control did not exhibit any colonization in hyphae. Overall, although refrigeration significantly enhanced the rate of hyphal colonization to roots, it remained considerably lower compared to that of spores. This may be due to the fact that the germ tubes of hyphae are much shorter than those of spores, and these short germ tubes cannot effectively contact and infect the roots. Furthermore, it may also be attributed to the weak infectivity of the hyphae itself.

AMF spores are structures that store fatty acids, the type of which is neutral lipid (mainly triacylglycerol) (Kobae et al., 2014). When AMF spores germinate, the energy and fatty acids required for the elongation of the germinating tube are derived from the spores, which convert the triacylglycerol (neutral lipid) into the membrane structure (phospholipid) in the germinating tube (Feng et al., 2020). The process of hyphae germination is not well understood and may be similar to that of spores, involving conversion of fatty acids from hyphae into those present in the germ tubes or through an unknown mechanism. Compared to 50 spores, the biomass and energy of 50 hyphae inoculated on each plate was less, and the germination rate of hyphae itself was lower than that of spores. Therefore, the colonization rate of hyphae was significantly lower than that of spores.

It has been confirmed that resting spores of AMF store abundant lipids, proteins, and glycogen, which are hydrolyzed to produce energy for maintaining metabolic activities, cell division, and DNA synthesis (Feng et al., 2020; Salmeron-Santiago et al., 2021; Kameoka and Gutjahr, 2022; Řezanka et al., 2023). When AMF spores initiate germination, lipids undergo degradation and are converted into trehalose or hexose through the glyoxylate cycle and gluconeogenesis pathway. Subsequently, these carbohydrates are metabolized via the glycolysis pathway, serving as precursors for the synthesis of cell walls, DNA, proteins, and other anabolic intermediates (Beilby and Kidby, 1982; Siqueira et al., 1985; Zhou et al., 2021; Kameoka and Gutjahr, 2022; Aparicio Chacón et al., 2023). A series of biological processes support the germination and hyphal growth of AMF. The mechanisms underlying the germination of AMF spores and hyphae remain elusive, as does the mechanism by which cold storage enhances their germination. Further investigations, such as RNA-Seq analysis incorporating various temperature treatments, different propagules, and diverse germination processes, are imperative for elucidating the molecular basis.

Our studies demonstrate that cold storage can effectively maintain and even enhance the germination ability of *in vitrol* culture AMF propagules over an extended period. In addition to refrigeration preservation, there were also reports of the use of cryopreservation, liquid nitrogen preservation, and other methods for preserving AMF. The storage methods for different AMF strains vary; certain strains were suitable for cold storage, some required freezing storage, and others could be stored at room temperature (Lalaymia et al., 2013, 2014). Lalaymia et al. (2014) also introduced a preservation method for *in vitro* culture AMF, which involved freezing AMF spores at -130°C in liquid nitrogen. Although the spores were able to germinate after 6 months of cryopreservation, the specific germination rate was not determined. Cryopreservation may represent a novel and efficient approach for long-term preservation of AMF, which needs further research and exploration. This study only explores one type of preservation condition of an AMF, while further exploration is needed to encompass various types of AMF and more efficient preservation methods for AMF inoculants. These aspects will be the focus of our future research endeavors.

## Conclusion

Our study further confirmed that spores, hyphae and root fragments were all effective propagules in AMF. Cold storage promoted the germination and growth of hyphae, and enhanced the mycorrhizal colonization ability of different propagules, however, compared with spores as inoculants, the mycorrhizal colonization of hyphae as inoculants was much less than spores. In terms of the effect of a single propagule, the colonization ability of hyphae was comparatively weaker than that of the other two propagules. The content quantity in a single hypha is evidently lower than that in a single spore, and our findings demonstrate that the length of hypha impacts its germination rate. In practical applications of AMF inoculants, there are numerous hyphae with varying lengths, and their colonization ability relies on both their number and length. Therefore, we cannot ignore the role of hyphae as propagules in inoculants. In addition, cold storage is an effective preservation method, which can maintain or even promote the colonization ability of AMF. Our work enhances the comprehension of AMF and contributes to the advancement of biological knowledge in this field. Moreover, our research holds significant potential for practical applications, as it enables the utilization of high-vitality AMF inoculants at any given time. Additionally, it eliminates wastage of AMF inoculants caused by prolonged culture at room temperature and reduces the time and energy expended on continuous subculture.

## Data Availability

The original contributions presented in the study are included in the article/**Supplementary Material**. Further inquiries can be directed to the corresponding authors.
