# Supplementary material for: Cold storage promotes germination and colonization of arbuscular mycorrhizal fungal hyphae as propagules
Source: Front Plant Sci. 2024 Nov 4;15:1450829. doi: 10.3389/fpls.2024.1450829 (PMC11570285; doi:10.3389/fpls.2024.1450829)
Supplement: Supplementary file 1 [file DataSheet1.pdf]

Supplementary Material

Supplementary Figures

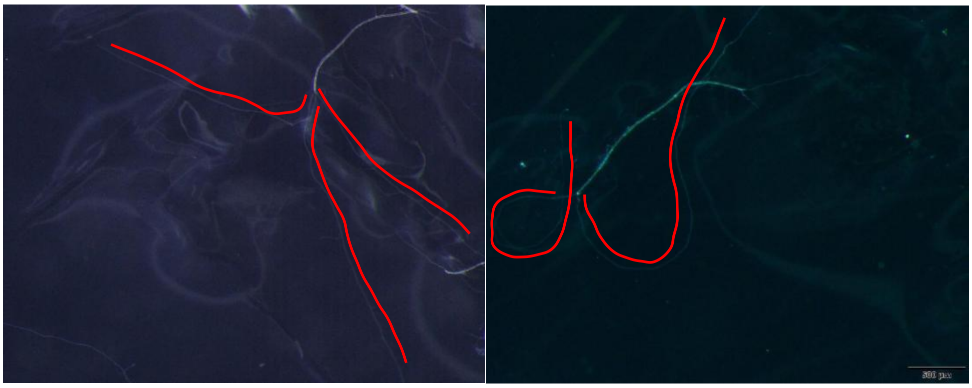

Supplementary Figure 1 the hyphae could produce two or three long germ tubes above 0.5 mm length at 6 months after cold-storage. Red lines indicate hyphal germ tubes.

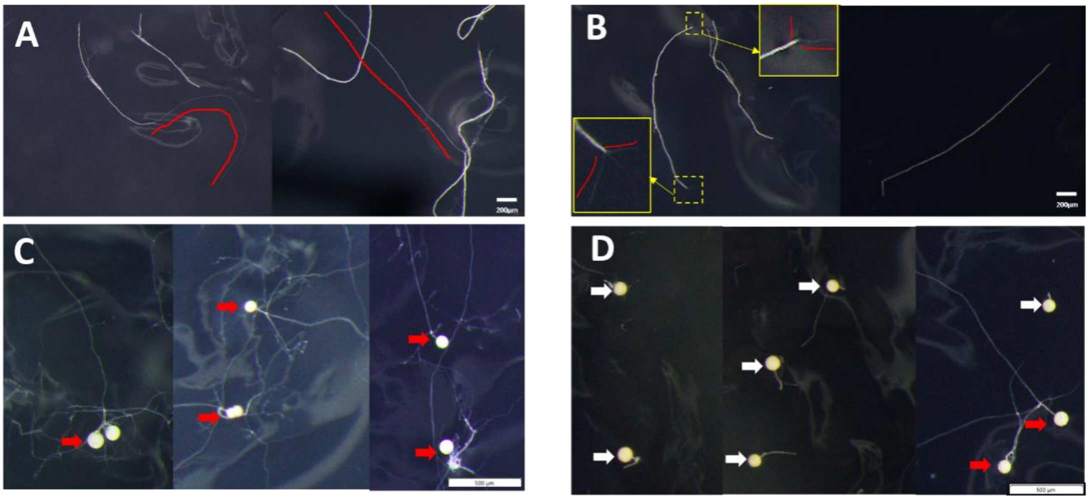

Supplementary Figure 2 Hyphae and spore germination in different treatments. The germination state of hyphae at 4℃ (A) and 25℃ (B) for 6 months. The germinated state of the spores at 4℃ (C) and 25℃ (D) for 6 months. Red lines indicate hyphal germ tubes, red arrows indicate germinated spores, and white arrows indicate non-germinated spores.

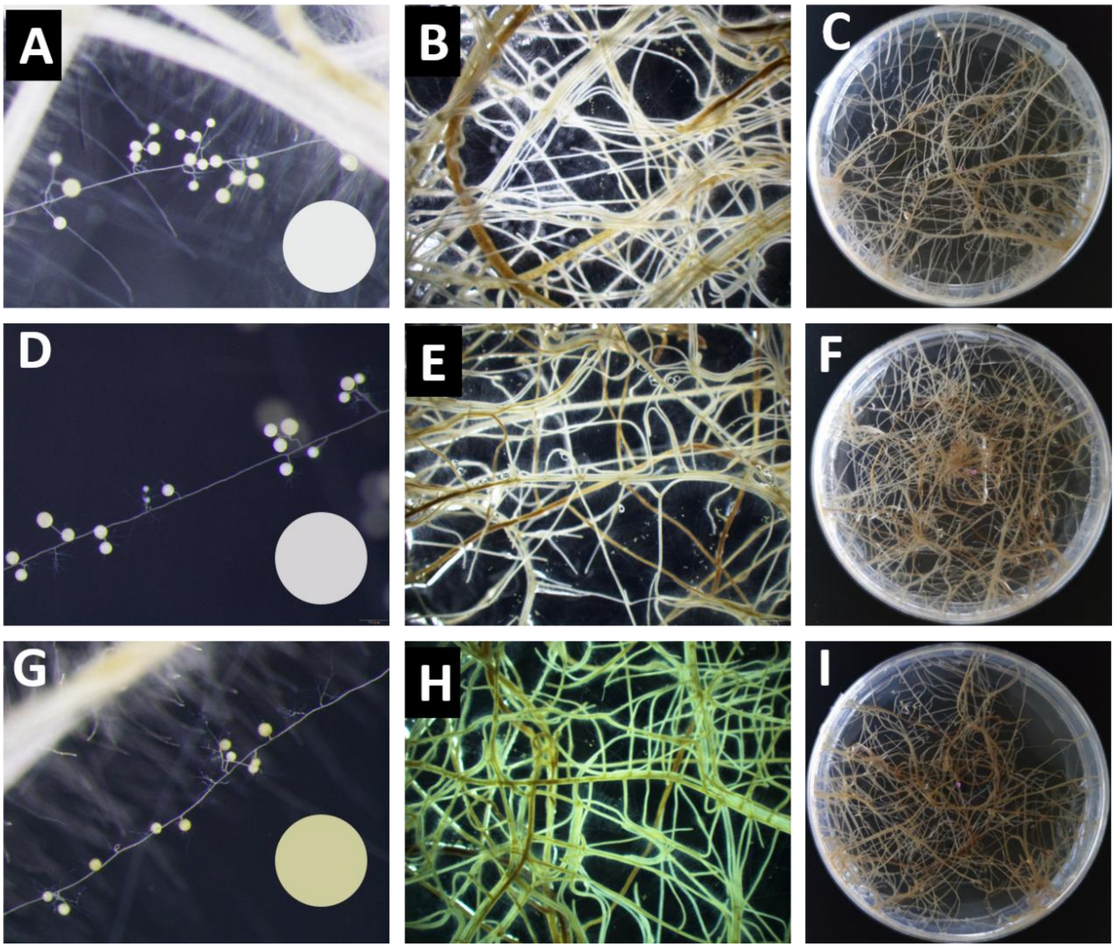

Supplementary Figure 3 Growth status of spores and roots in different treatments. Growth status of spores, roots and cultures on whole plates at 25°C for 0 months (A-C), 4°C for 6 months (D-F), and 25°C for 6 months (G-I). The circles in the lower right corners of figure A, D, and G indicate spore color.
